# Changes in Polar Metabolites Content during Natural and Methyl-Jasmonate-Promoted Senescence of *Ginkgo biloba* Leaves

**DOI:** 10.3390/ijms23010266

**Published:** 2021-12-27

**Authors:** Marcin Horbowicz, Joanna Szablińska-Piernik, Justyna Góraj-Koniarska, Kensuke Miyamoto, Junichi Ueda, Marian Saniewski

**Affiliations:** 1 Department of Plant Physiology, Genetics and Biotechnology, University of Warmia and Mazury, Oczapowskiego 1a, 10-719 Olsztyn, Poland; Joanna.Szablinska@uwm.edu.pl; 2 Research Institute of Horticulture, Konstytucji 3 Maja 1/3, 96-100 Skierniewice, Poland; Justyna.Goraj@inhort.pl (J.G.-K.); Marian.Saniewski@inhort.pl (M.S.); 3 Faculty of Liberal Arts and Sciences, Osaka Prefecture University, 1-1 Gakuen-cho, Naka-ku, Sakai, Osaka 599-8531, Japan; miyamoto@las.osakafu-u.ac.jp; 4 Department of Biological Science, Graduate School of Science, Osaka Prefecture University, 1-1 Gakuen-cho, Naka-ku, Sakai, Osaka 599-8531, Japan; ueda@b.s.osakafu-u.ac.jp

**Keywords:** *Ginkgo biloba*, methyl jasmonate, leaf senescence, amino acids, organic acids, carbohydrates, cyclitols

## Abstract

The present study clarified changes in the contents of polar metabolites (amino acids, organic acids, saccharides, cyclitols, and phosphoric acid) in leaf senescence in *Ginkgo biloba* with or without the application of methyl jasmonate (JA-Me) in comparison with those in naturally senescent leaf blades and petioles. The contents of most amino acids and citric and malic acids were significantly higher in abaxially, and that of *myo*-inositol was lower in abaxially JA-Me-treated leaves than in adaxially JA-Me-treated and naturally senescent leaves. The levels of succinic and fumaric acids in leaves treated adaxially substantially high, but not in naturally senescent leaves. In contrast, sucrose, glucose, and fructose contents were much lower in leaf blades and petioles treated abaxially with JA-Me than those treated adaxially. The levels of these saccharides were also lower compared with those in naturally senescent leaves. Shikimic acid and quinic acid were present at high levels in leaf blades and petioles of *G. biloba*. In leaves naturally senescent, their levels were higher compared to green leaves. The shikimic acid content was also higher in the organs of naturally yellow leaves than in those treated with JA-Me. These results strongly suggest that JA-Me applied abaxially significantly enhanced processes of primary metabolism during senescence of *G. biloba* compared with those applied adaxially. The changes in polar metabolites in relation to natural senescence were also discussed.

## 1. Introduction

*Ginkgo biloba* (L.), the only living species in the Ginkgoaceae family, is one of the most commercialized medicinal plants, as its leaf extract is currently the best-selling herbal product, which is believed to contain ingredients that improve memory, benefit Alzheimer’s patients, and increase blood circulation. The leaf extract contains many constituents, such as terpene trilactones (TTL: ginkgolides and bilobalides, unique compounds found only in *G. biloba*), flavonoids, phenolic acids, amino acids, saccharides, and various organic acids [1,2,3,4,5,6,7,8,9,10]. *G. biloba* is also planted worldwide as an ornamental tree for its magnificent golden color in autumn foliage and for its resistance to urban conditions [11].

Phytohormones play a key role in the regulation of various developmental processes in plants, as well as in the integration of environmental signals with various physiological processes. Senescence is the last drastic physiological phenomenon occurring in all plants and at all stages of the plant life cycle, and defined as changes that lead sooner or later to the death of an organism or part of it. Leaf senescence in deciduous trees is most conspicuous due to the coloration of the foliage. The process of leaf senescence is well known and is related to the loss of chlorophyll and rapid decomposition of proteins, accompanied by an increase in amino acids, after which leaves finally abscise [12].

A wide range of plant growth regulators, including phytohormones, are believed to be involved in the regulation of all stages of the plant life cycle. Among them, jasmonic acid (JA) and methyl jasmonate (JA-Me) (referred to as jasmonates, JAs), which were first isolated from wormwood (*Artemisia absinthium*) and *Cleyera ochnacea* as senescence-promoting substances [13], have been recognized as being crucial for the regulation of senescence in plants, and many papers have been published on the role of JAs in the senescence mechanism of plant leaves [14,15,16,17,18,19,20,21,22,23,24,25]. Conclusively, it has been shown that many aging-related genes in *Arabidopsis* are regulated by JAs [26,27,28]. JAs have also been demonstrated to be involved in triggering various biochemical and physiological processes in plants. For example, they play key crucial roles in the pathways of the secondary metabolites, such as phytoalexins, volatile terpenes [17], sesquiterpene trilactones [29,30], flavonoids [31], lignins [32,33], and phenolic acids [34,35]. In *Arabidopsis thaliana*, JA-Me has been demonstrated to increase or decrease the levels of organic acids, carbohydrates, and some of amino acids [36].

In our previous study, it was shown that JA-Me applied in lanolin paste to the abaxial side in the middle part of the leaf blade of *G. biloba* in early September remarkably promoted leaf senescence by stimulating chlorophyll degradation in all senescence stages of the life cycle, although JA-Me applied to the adaxial sides of leaves induced leaf senescence to a minor extent [37]. A comprehensive analyses of phytohormones revealed that the application of JA-Me on the abaxial side of the leaf blade significantly increased its level as well as that of JA in the leaf blade and petiole. Moreover, the level of endogenous abscisic acid (ABA) significantly increased during natural and JA-Me-induced leaf aging, suggesting that a close interaction between JAs and ABA plays a regulatory role in leaf senescence in *G. biloba*.

Furthermore, we have found that levels of the major TTLs, ginkgolides B and C, have been demonstrated to be significantly higher in naturally senesced yellow leaves compared with green leaves, and the application of JA-Me slightly but substantially increased the levels of TTLs in leaf blades, irrespective of the application side [38]. Some flavonoids and phenolic acids also showed changes in content related to maturation or senescence. The levels of quercetin, rutin, quercetin-4-glucoside, apigenin, and luteolin were dependent on the site of JA-Me application, and the application of JA-Me increased ferulic acid and *p*-coumaric acid esters in the petiole but decreased the levels of these compounds in the leaf blade. In general, JA-Me modified the accumulation of TTLs, flavonoids, and phenolic acids, as well as caused leaf senescence. On the other hand, JA-Me- and aging-related metabolic changes in endogenous levels of polar metabolites such as soluble sugars, free amino acids, and organic acids in *G. biloba* leaves have not been demonstrated well. It is considered important to know the metabolic changes in these polar compounds in *G. biloba* leaves in relation to leaf senescence.

The aim of this study was to examine changes in polar metabolite levels in the leaf blade and petiole of *G. biloba* during natural and JA-Me-induced senescence. This study is a continuation of our previous ones in this area [37,38].

## 2. Results

### 2.1. Effects of JA-Me on the Contents of Free Amino Acids

**Figure 1:**
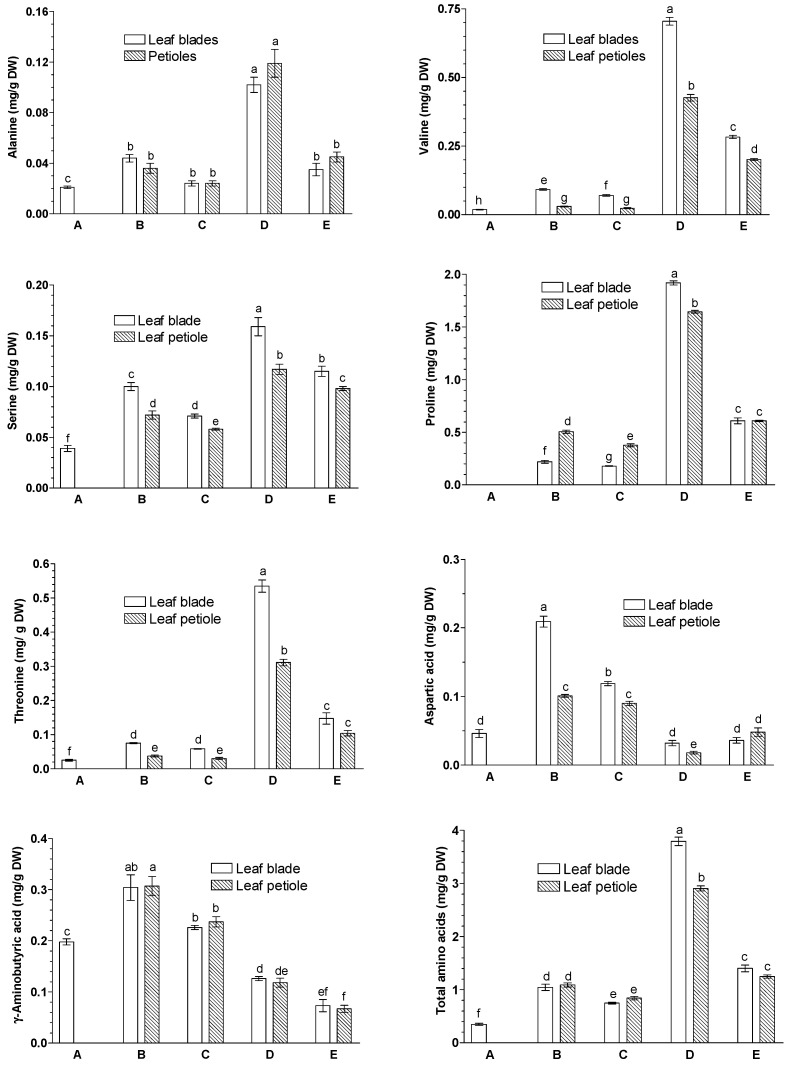
Effects of methyl jasmonate (JA-Me) applied to the adaxial or abaxial side of the leaf blade on the contents (mg/g DW) of free amino acids in leaf blades and petioles of *G. biloba*. Mean results ± SD followed by the same letter were not significantly different (*p* < 0.05) according to Tukey’s test. A—initial stage, leaf samples collected on 9 September; B—control (lanoline), leaf samples collected on 30 September; C—JA-Me applied to the adaxial side, leaf samples collected on 30 September; D—JA-Me applied to the abaxial side, leaf samples collected on 30 September; E—naturally senesced leaf, samples collected on 20 October.

The following free amino acids were identified and their endogenous levels were determined in leaf blades and petioles of *G. biloba*: alanine (Ala), leucine (Leu), valine (Val), serine (Ser), proline (Pro), threonine (Thr), aspartic acid (Asp), and gamma (γ)-aminobutyric acid (GABA), which is a non-protein amino acid synthesized via conversion of glutamate by glutamate decarboxylase (Figure 1; Supplementary Table S1). Among them, Pro was present at the highest level in leaf blades and petioles.

In leaf blades, at the initial time, the free amino acid contents were quite low. During the natural senescence process, the contents of free amino acids detected in leaf blades substantially changed, with the levels of Ala, Leu, Ser, Val, Thr, and Pro increasing and that of GABA decreasing. Three weeks after the application of JA-Me, irrespective of the side of treatment (adaxial or abaxial), the contents of all free amino acids detected increased, except leucine. In particular, there were marked increases in amino acid contents in leaf blades in which JA-Me was applied to the abaxial side of the leaves. Leu was present only in leaf blades of naturally senescing yellow leaves and in leaves senescing under JA-Me applied to the abaxial side. Similar changes in amino acid content under the influence of JA-Me, regardless of the place of its application, were also observed in petioles.

As shown in Supplementary Figure S1, the composition of free amino acids in leaf blades and petioles was significantly altered by natural senescence or by senescence under JA-Me applied to the abaxial side of the leaf, with an increased ratio of Pro to GABA. These results suggest that senescence process substantially affects amino acid metabolism in *G. biloba* leaves.

### 2.2. Effects of JA-Me on the Contents of Organic Acids and Phosphoric Acid

**Figure 2:**
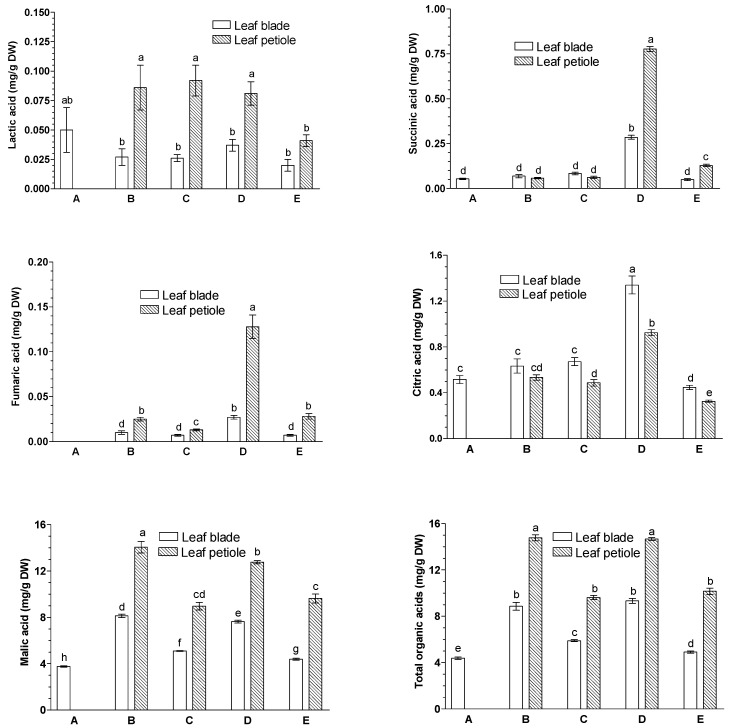
Effects of methyl jasmonate (JA-Me) applied to the adaxial and abaxial sides of the leaf blade on the contents (mg/g DW) of organic acids in leaf blades and petioles of *G. biloba*. Mean results ± SD followed by the same letter were not significantly different (*p* < 0.05) according to Tukey’s test. A—initial stage, leaf samples collected on 9 September; B—control (lanoline), leaf samples collected on 30 September; C—JA-Me applied to the adaxial side, leaf samples collected on 30 September; D—JA-Me applied to the abaxial side, leaf samples collected on 30 September; E—naturally senesced leaf, samples collected on 20 October.

The following organic acids were identified in the leaf blade and petiole of *G. biloba*: lactic, citric, succinic, fumaric, and malic acids, as well as inorganic phosphoric acid (Figure 2; Table 1).

Among the organic acids present in the leaf organs of *G. biloba*, the contents of succinic, fumaric, and citric acids were significantly increased in the leaf blades of leaves treated with JA-Me on the abaxial side compared with JA-Me treatment on the adaxial side and control leaves (Figure 2). In contrast, malic acid content was only slightly higher in the leaf blades of *G. biloba* after JA-Me treatment on the abaxial side compared with JA-Me treatment on the adaxial side.

Almost the same tendency in the content of organic acids determined in the leaf blade was observed in the petiole. These results suggest that JA-Me influences the circulation of the mentioned organic acids in *G. biloba* leaves. However, the content of lactic acid was not significantly dependent on the JA-Me application site, while the content of phosphoric acid was almost twice as high in leaf blades and petioles after abaxial JA-Me application compared with adaxial, but was the same as that of the control leaves.

### 2.3. Effects of JA-Me on the Contents of Saccharides, Cyclitols, and Intermediates in the Aromatic Compound Synthesis

**Figure 3:**
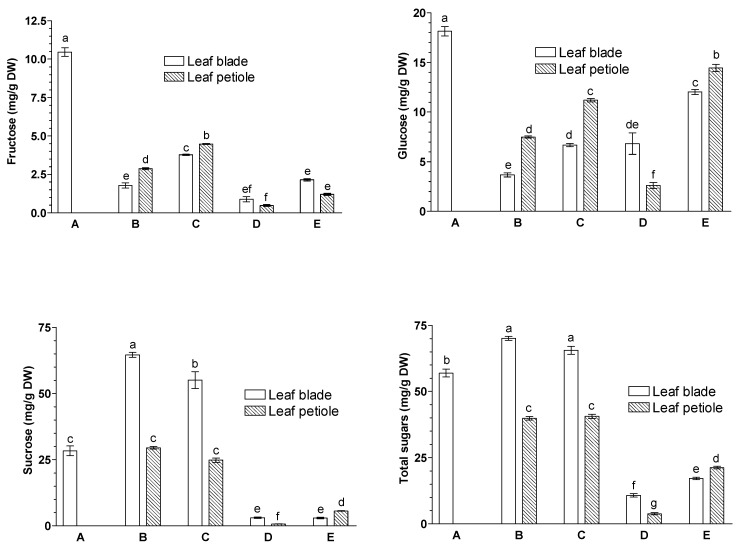
Effects of methyl jasmonate (JA-Me) applied to the adaxial and abaxial sides of leaf blade on the contents (mg/g DW) of sugars in leaf blades and petioles of *G. biloba*. Mean results ± SD followed by the same letter were not significantly different (*p* < 0.05) according to Tukey’s test. A—initial stage, leaf samples collected on 9 September; B—control (lanoline), leaf samples collected on 30 September; C—JA-Me applied to the adaxial side, leaf samples collected on 30 September; D—JA-Me applied to the abaxial side, leaf samples collected on 30 September; E—naturally senesced leaf, samples collected on 20 October.

The following soluble sugars were identified and their endogenous contents were determined in leaf blades and petioles of *G. biloba* leaves, namely fructose (Fru), glucose (Glc) and sucrose (Suc) (Figure 3), whereas trace amounts of galactose were present (data not shown).

In the blades of *G. biloba* leaves, the contents of Fru and Glc were dramatically reduced during aging and the natural senescence process (Figure 3). On the other hand, the application of JA-Me to the abaxial side of the leaf dramatically reduced the contents of Fru and Suc compared to natural senescence, although application to the adaxial side of the leaf showed little effect. In contrast, Glc content was increased by the application of JA-Me irrespective of the application sides as compared to the control. In control leaves of *G. biloba*, the proportion of Suc was quite high, accounting for more than 80% of all soluble sugars (Supplementary Figure S2). Application of JA-Me to the abaxial side of the leaf significantly reduced the proportion of Suc (less than 20%) and increased the proportion of Glc (about 60%) relative to the naturally senescent one, whereas application of JA-Me to the adaxial side had no effect on this. This suggests that sucrose-related sugar metabolism was quite sensitive to leaf senescence.

**Figure 4:**
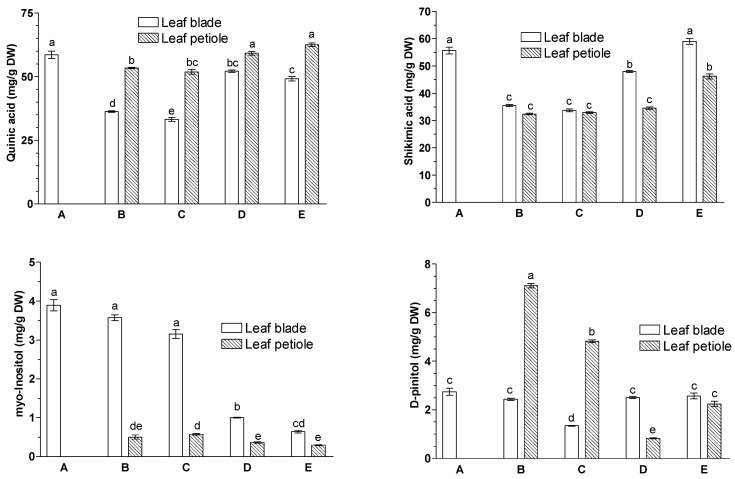
Effects of methyl jasmonate (JA-Me) applied to the adaxial and abaxial sides of the leaf blade on the contents (mg/g DW) of cyclohexitols in leaf blades and petioles of *G. biloba*. Mean results ± SD followed by the same letter were not significantly different (*p* < 0.05) according to Tukey’s test. A—initial stage, leaf samples collected on 9 September; B—control (lanoline), leaf samples collected on 30 September; C—JA-Me applied to the adaxial side, leaf samples collected on 30 September; D—JA-Me applied to the abaxial side, leaf samples collected on 30 September; E—naturally senesced leaf, samples collected on 20 October.

Of cyclitols, the *myo*-inositol content decreased in leaf blades and petioles in leaves treated with JA-Me on the abaxial side as compared to the control leaves (Figure 4). In contrast, the d-pinitol content increased slightly in leaf blades and decreased significantly in the petioles of leaves treated with JA-Me on the abaxial side as compared to the control leaf. Almost the same effect of JA-Me in leaf blades was observed in petioles of *G. biloba* leaves.

Quinic acid and shikimic acid are key intermediates in the biosynthesis of aromatic compounds in plants. Both are polyhydroxyl derivatives of cyclohexane containing an additional carboxyl group. Shikimic acid and quinic acid were present in high levels in the leaf blades of *G. biloba*, but JA-Me did not affect their contents, either after its application on the adaxial or abaxial side of the leaves. The contents of both acids were correlated with each other. Pearson’s coefficients were 0.879 and 0.847 for leaf blades and petioles, respectively, and this correlation was highly significant (<0.001).

### 2.4. Principal Component Analysis

**Figure 5:**
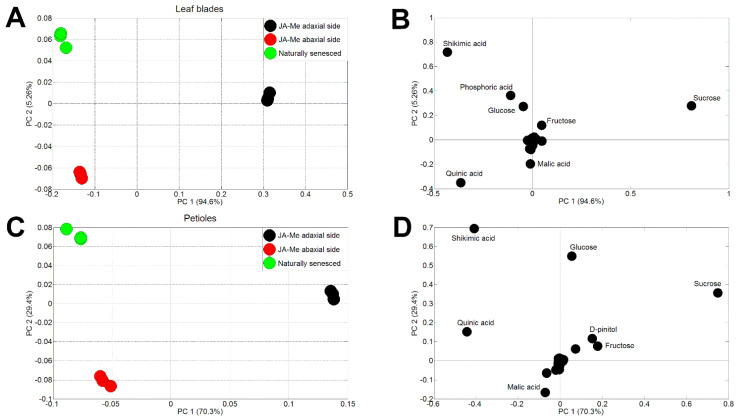
PCA scores (**A**,**C**) and loadings (**B**,**D**) of metabolic profiles of leaf blades and petioles of *G. biloba* treated with methyl jasmonate (JA-Me) on the adaxial (black dots) and abaxial sides (red dots), as well as naturally senescent ones (green dots).

Principal component analysis (PCA) analysis applied to the entire metabolome dataset (normalized data) showed overall variation in the metabolite levels relative to the leaf senescence factors examined (Figure 5A–D). High PC1 scores for both leaf blades (94.6%) and petioles (70.3%) indicated that there were significant differences in metabolite composition among each leaf senescence factor (Figure 5A,C). The highest differences were evident between the metabolic profiles of JA-Me-treated leaves on the adaxial and abaxial sides. The low PC2 values (5.26 and 29.4%) indicated that the metabolic profiles of leaves and petioles treated with JA-Me on the abaxial side were more similar to those of naturally senesced leaves (Figure 5A,C). The PCA loading plots show the major contributions of the identified metabolites to the different factors of leaf senescence. For both leaf blades and petioles, Suc, Glc, shikimic acid, and quinic acid were the metabolites most responsible for this variation (Figure 5B,D).

## 3. Discussion

Polar metabolites such as amino acids, saccharides, and organic acids are some of the most important constituents from a biological point of view as the basic structural units of proteins and cell walls and as sources of energy for the biosynthesis of important secondary metabolites. As previously described, the application of JA-Me to the abaxial (lower) side of green *G. biloba* leaves significantly induced their senescence and the degradation of chlorophylls, while control leaves remained green [37]. JA-Me applied on the adaxial side was less effective in inducing leaf senescence compared to that on the abaxial side. JA-Me applied on the abaxial sides of leaves also substantially induced increases in the pharmaceutically valuable secondary metabolites, such as ginkgolides, bilobalide, flavonoids, and phenolic acids in leaf blades of *G. biloba* [38]. Thus, it is worthy to clarify the changes in endogenous polar metabolites in relation to senescence promoted by JA-Me application, as well as natural senescence in *G. biloba* leaves.

Of the free amino acids, Ala, Leu, Val, Ser, Pro, Thr, Asp, and GABA were identified in leaf blades and petioles of *G. biloba* leaves, with the levels of GABA being much higher than the other amino acids identified (Figure 1). Similar to our current study, Carratú et al. [39] determined Ala, Leu, Val, Ser, Pro, Asp, arginine, Asn, Gln, glutamic acid (Glu), Thr, methionine (Met), phenylalanine (Phe), isoleucine (Ile), and GABA in dried tissue samples of *G. biloba*, while Phe was present only in trace amounts. In turn, Hodisan et al. [40] identified Ala, glycine (Gly), isoleucine (Ile), Leu, Pro, Asp, phenylalanine (Phe), tyrosine (Tyr), and Glu in *G. biloba* leaf extracts. Yao et al. [7] demonstrated the presence of Phe, Leu, Ile, Val, Thr, lysine (Lys), and GABA, and the contents of these amino acids were obviously different, with the total content of all the target compounds ranging from 1.4 to 14.79 mg/g DW. These results suggest that free amino acids are obviously different qualitatively and quantitatively in *G. biloba* leaves, which might depended on the age of the trees, the season they were harvested, and various growing environments.

Leaf senescence is crucial for plant fitness as a process of nutrient relocation from leaves to other organs, with amino acids being partially recovered and used to synthesize specific proteins needed under nutrient-deficient conditions during plant senescence [12,41]. This seems to be a major cause of large changes in their contents. The senescence process of a tissue often includes proteolysis, resulting in an increase in free amino acids [42]. It was previously shown that during the senescence of detached oat leaves there was an increase in free amino acid levels, while attached oat leaves showed a decrease in its content during senescence [43]. In *Arabidopsis*, both attached and detached leaves showed gradual increases in amino acid contents during senescence [43]. In this context, amino acid regulation and transport are critical for plant adaptation, development, and defense [44]. The mechanisms underlying the regulation of amino acid metabolism in plants are largely unknown, but are certainly very complex.

As shown in Figure 1 and Supplementary Figure S1, the application of JA-Me on the abaxial side of the leaf of *G. biloba* substantially increased the contents of Ser, Val, Thr, and Pro in leaf blades and the petioles of *G. biloba,* with the amino acid composition in senescent leaves after JA-Me application being compatible to that of naturally senescent leaves. Hendrawati et al. [36] documented that JA-Me caused increased contents of Trp, Val, Thr, and Ala and an decrease in Gln 24 h after treatment with *Arabidopsis thaliana*. It has also been reported that JA-Me application to the leaves increased amino acids in leaves of grapes [45,46] and tobacco [47]. Further studies on the effects of JA-Me on protein metabolism, synthesis, and degradation of proteins in *G. biloba* will be required.

Amino acid metabolism is tightly linked to carbohydrate metabolism as well as to the demand for protein synthesis and secondary metabolism (e.g., biosynthesis of phenylpropanoids, alkaloids, and other secondary metabolites) [44]. Carbohydrates, such as Fru, Glc, and Suc, were found in initial *G. biloba* leaves, with Suc being the major sugar (Figure 3; Supplementary Figure S2). JA-Me extremely decreased Suc and Fru contents in leaf blades, as natural senescence did, whereas it had little effect on the Glc content. In rice leaves treated with JA-Me, a similar reduction in sucrose content was observed [48]. Additionally, it was previously reported that JA reduced the starch concentration in leaves of *Populus* [49], and treatment with JA-Me reduced the pool of soluble sugars in *Nicotiana tabacum* leaves [47]. JA also reduced leaf sugars in *Brassica oleracea* [50]. Furthermore, changes in the metabolism of cell wall polysaccharides during senescence induced by JA-Me application has been demonstrated in oat leaves [51].

During leaf senescence, sugars often accumulate. On the other hand, the accumulation of sugars induced leaf senescence [52]. As shown in Figure 3, the levels of sucrose in leaves was substantially reduced in senescent JA-Me *G. biloba* leaves. These results suggest that leaf senescence is possibly crucial for plant fitness, as sugar relocation occurs from senescent leaves to other organs. Further studies on the mechanism of JA-Me in regulating sugar pools, especially sucrose, will be necessary in relation to invertase activity in future.

GABA, a non-protein amino acid, is synthesized *via* conversion of glutamate by glutamate decarboxylase, which is induced by stress conditions. Hijaz and Killiny [53] documented that the levels of endogenous GABA, succinic acid, and fumaric acid were rapidly increased in *Citrus aurantifolia* leaves treated with GABA. The results obtained indicated that GABA was uptaken by the leaf, metabolized to succinic acid, and then entered the tricarboxylic acid cycle (TCA). As presented in Figure 1 and Figure 2, JA-Me applied to the abaxial sides of *G. biloba* leaves decreased the level of GABA, but increased the levels of succinic acids and fumaric acids, suggesting that GABA is metabolized to these organic acids. Gene expression analysis of citrate metabolism showed that the high citrate accumulation could be attributed to the low GABA content and was partly due to TCA cycle blockade due to low expression levels of mitochondrial aconitase [54]. Moreover, it was found that the insecticidal activity of *G. biloba* extracts can be attributed to their effects on GABA receptors [55].

Abscisic acid (ABA) and JAs have been reported to play crucial roles in plant adaptation to stress conditions, and these phytohormones are involved in inducing stomatal closure as well [56,57]. Since stomata play a key role in the assimilation of carbon dioxide and transpiration, their function directly affects the efficiency of photosynthesis [58]. In our previous study, it was shown that the application of JA-Me to the abaxial side of *Ginkgo* leaves resulted in extremely induced leaf senescence [37]. This was accompanied by a rapid increase in ABA content. Furthermore, it was shown that ABA levels were significantly higher during JA-Me-induced senescence than in natural senescence. The increase in ABA content probably induced stomata closure, resulting in inhibition of photosynthesis. As a result, there were large decreases in sucrose and fructose contents in the leaf blades of *G. biloba* (Figure 3). Wei et al. [59] also found during leaf senescence of *G. biloba* that the decrease in photosynthesis was mainly due to decreased stomatal conductance and degradation of the photosynthetic apparatus. Additionally, the decrease in JA-mediated sugars may have been the result of reductions in primary metabolites and an increased secondary metabolism [60,61,62,63]. It is known that stomata in *G. biloba* leaves occur only in the abaxial epidermis [64,65], and this localization of stomata is involved in the differential effectiveness of JA-Me application to the adaxial side or abaxial sides.

Organic acids, namely oxalic, quinic, malic, and shikimic acids, were also identified and quantified, with quinic acid being the most abundant organic acid in the leaves of the *Ginkgo* plant [66]. Quinic acid and shikimic acid, which are polyhydroxyl derivatives of cyclohexane containing an additional carboxyl group, are key intermediates in the biosynthesis of aromatic compounds in plants. Further, *myo*-inositol is a cyclitol which is important for plant growth and development, because it is a factor needed for phosphorus storage in seeds. Moreover, it is involved in cell wall biosynthesis and the production of stress-related molecules, and is an essential component of a signaling pathway in plants called the Pi signaling pathway. It is also believed to play a central role in controlling auxin action and transport [67]. The isomerization and methylation of *myo*-inositol produces various o-methyl inositols, including d-pinitol, which are involved in plant stress responses [67]. The plant cells contain several indole-3-acetic acid conjugates, including indole-3-acetic acid-*myo*-inositol, which enable storage of excess auxin [68,69].

As shown in Figure 4, *G. biloba* leaves contains relatively high amounts of shikimic acid. In the pharmaceutical industry, shikimic acid is used to produce Tamiflu (Oseltamivir) used against influenzas A and B [70], and *G. biloba* tissue can be used to extract this compound. The shikimate pathway links carbohydrate metabolism to the biosynthesis of aromatic amino acids essential for protein biosynthesis and numerous secondary metabolites in plants [71]. In a sequence of several metabolic steps, phosphoenolpyruvate and erythrose 4-phosphate are converted to chorismate, a precursor of Phe, Tyr, and Trp and many secondary metabolites such as phenolic acids and flavonoids [71,72,73,74,75]. Among the intermediates of the shikimate pathway, quinate can be formed directly from shikimate or 3-dehydroquinate. Guo et al. [76] showed that in poplar, quinate and shikimate metabolism is associated with the same set of genes. This may indicate that there is a close correlation between the accumulation of both metabolites in the plant. The close correlation between the two acids was demonstrated in the present study *via* their contents in leaf blades and petioles.

## 4. Materials and Methods

### 4.1. Plant Material

A twelve-year-old *G. biloba* tree growing in Skierniewice, Poland was used for the study. The gender of the tree used in this study was not determined. Treatments were conducted on different branches of one tree, and 20–30 leaves were used for each treatment. Green leaves were treated with methyl jasmonate (JA-Me) at a concentration of 0.5% (*w/w*) in lanolin paste containing 30% water (*w/w*). It was applied as a 2–3 mm wide strip in the central part of the leaf on the adaxial (upper) or abaxial (lower) side, and pure lanolin with 30% water content was used as a control. The JA-Me treatment was performed on 9 September 2017. Three weeks later JA-Me-treated and control leaves were collected, freeze-dried, and analyzed after fine milling. At that time, control leaves and leaves treated with JA-Me on the adaxial side were green, and leaves treated with JA-Me on the abaxial side were yellow. To compare JA-Me-induced senescence with the natural senescence process, yellow leaves were collected on 20 October and also analyzed. The weather conditions during the experiment are shown in Supplementary Table S1. More details of the experimental conditions along with pictures of control and JA-Me-treated *G. biloba* leaves are presented in our previous paper [37].

### 4.2. Extraction of Polar Metabolites

Extraction of polar metabolites (free amino acids, organic acids, saccharides, and others) was performed according to the method described by Szablińska-Piernik and Lahuta [77]. Briefly, polar metabolites were extracted from ground, freeze-dried tissues (40–45 mg) by heating at 70 °C for 30 min with continuous shaking at 500 rpm using 1 mL of methanol/water mixture (1:1, *v*/*v*) containing 100 μg of ribitol (internal standard). The mixture was centrifuged under 20,000× *g* at 4 °C for 20 min, then 400 μL of cold chloroform was added to 600 μL of the supernatant to remove non-polar compounds. After shaking at 1300 rpm for 15 min with Vortex Genie 2 (Scientific Industries, Bohemia, NY, USA) and centrifugation under 20,000× *g* at 4 °C for 10 min, 200 μL of the upper layer (methanol/water polar fraction) was concentrated in a vacuum rotary evaporator to dryness.

### 4.3. Derivatization of Polar Metabolites

Chemical reactions to obtain volatile derivatives of polar metabolites were carried out according to the method used by Lisec et al. [78]. Dry samples were derivatized in two steps by applying 40 μL of O-methoxamine hydrochloride (20 mg/mL pyridine) with heating at 37 °C for 75 min, with continuous shaking at 500 rpm using a shaker (Thermo- MS-100, Hangzhou Allsheng Instruments, Hangzhou, China), followed by the addition of 160 μL of a mixture of N-methyl-N-(trimethylsilyl) trifluoroacetamide and pyridine (1:1, *v*/*v*) and heating at 70 °C for 30 min.

### 4.4. Gas Chromatographic Analyses of Polar Metabolites

The metabolite derivatives obtained in this way were analyzed using a ZEBRON ZB5-MSi Guardian (5% phenyl—95% dimethylpolysiloxane) column (30 m length, ϕ 0.25 mm, 0.25 μm film thickness, Phenomenex, Torrance, CA, USA) using a GC-2010 gas chromatograph (Shimadzu, Japan) equipped with a flame ionization detector and a GC-2010 coupled to a quadrupole mass spectrometry analyzer (GCMS-QP2010 Plus, Shimadzu, Kyoto, Japan). Details of the analyses have been described previously [77]. Metabolites were identified by retention time (RT) and mass spectra compared to the original standards (Sigma-Aldrich, Saint Louis, MO, USA) and data contained in the NIST 05 database (National Institute of Standards and Technology, Gaithersburg, MD, USA).

### 4.5. Principal Component Analysis

Normalized data were considered using a multivariate statistics analysis (principal component analysis, PCA), which was performed in three replicates using COVAIN, a MATLAB toolbox including a graphical user interface (MATLAB version 2013a; Math Works, Natick, MA, USA) [79].

### 4.6. Statitistics

Analyses of the *G. biloba* tissues were performed in three replicates. Analysis of variance (one way ANOVA) and Tukey’s post hoc test were used to check the significance of the differences. These calculations and the Pearson’s correlation coefficients were performed using Statistica 12PL software (StatSoft, Tulsa, OK, USA). The results are shown as means ± standard deviation (SD).

## Figures and Tables

**Table 1:** Effects of methyl jasmonate (JA-Me) applied to the adaxial and the abaxial sides of the leaf blade on the contents (mg/g DW) of phosphoric acid in leaf blades and petioles of *G. biloba*. Mean results ± SD followed by the same letter were not significantly different (*p* < 0.05) according to Tukey’s test.

Organ	Initial Day (9 September)	Control (lanolin) (30 September)	Adaxial Treatment with JA-Me (30 September)	Abaxial Treatment with JA-Me (30 September)	Naturally Senescent (20 October)
Leaf blade	22.95 ± 0.55 ^a^	8.41 ± 0.06 ^e^	4.09 ± 0.04 ^f^	8.70 ± 0.35 ^de^	18.72 ± 1.05 ^b^
Leaf petiole	-	7.44 ± 0.13 ^e^	7.63 ± 0.05 ^e^	11.26 ± 0.11^c^	9.62 ± 0.18 ^d^

## Data Availability

The data presented in this study are available in this article.

## Supplementary Materials

The following are available online at https://www.mdpi.com/article/10.3390/ijms23010266/s1.

## Sample Availability

Samples of the compounds are not available from the authors.
