# Supplementary material for: Changes in Polar Metabolites Content during Natural and Methyl-Jasmonate-Promoted Senescence of Ginkgo biloba Leaves
Source: Int J Mol Sci. 2021 Dec 27;23(1):266. doi: 10.3390/ijms23010266 (PMC8745189; doi:10.3390/ijms23010266)
Supplement: Supplementary file 1 [file ijms-23-00266-s001.zip › ijms-1503024-Supplementary Materials.pdf]

Table S1. Primary weather conditions in Skierniewice between September 9, 2017 and October 20, 2017.

Day	Temp. min. (°C)	Temp. max. (°C)	Cloudiness
09.9.2017	14	24	Cloudless
10.9.2017	17	21	Partly cloudy
11.9.2017	14	22	Lightly cloudy
12.9.2017	16	17	Rain; Lightly cloudy
13.9.2017	14	18	Lightly cloudy
14.9.2017	15	17	Lightly cloudy
15.9.2017	9	15	Rain; Lightly cloudy
16.9.2017	8	16	Rain; Lightly cloudy
17.9.2017	11	13	Rain; total cloudiness
18.9.2017	9	16	Lightly cloudy
19.9.2017	5	17	Lightly cloudy; Cloudless
20.9.2017	11	12	Lightly cloudy
21.9.2017	9	12	Rain; total cloudiness
22.9.2017	11	13	Rain; Partly cloudy
23.9.2017	11	15	Rain; total cloudiness
24.9.2017	12	16	Rain; Partly cloudy
25.9.2017	11	16	Rain; Partly cloudy
26.9.2017	11	18	Rain; total cloudiness
27.9.2017	10	18	Cloudless
28.9.2017	8	18	Cloudless
29.9.2017	5	13	Lightly cloudy
30.09.2017	7	16	Cloudless
01.10.2017	4	16	Cloudless
02.10.2017	3	18	Cloudless
03.10.2017	10	12	Rain; cloudy
04.10.2017	9	13	Lightly cloudy
05.10.2017	7	12	Rain; cloudy
06.10.2017	7	11	Rain; Partly cloudy
07.10.2017	8	12	Rain; Lightly cloudy
08.10.2017	4	11	Rain; Partly cloudy
09.10.2017	3	11	Lightly cloudy
10.10.2017	6	12	Lightly cloudy; Rain
11.10.2017	11	14	Rain; Partly cloudy
12.10.2017	10	17	Rain; Partly cloudy
13.10.2017	9	14	Lightly cloudy
14.10.2017	5	16	Partly cloudy
15.10.2017	10	18	Partly cloudy
16.10.2017	8	21	Cloudless
17.10.2017	7	22	Cloudless
18.10.2017	9	21	Cloudless
19.10.2017	7	18	Cloudless
20.10.2017	10	12	Cloudy

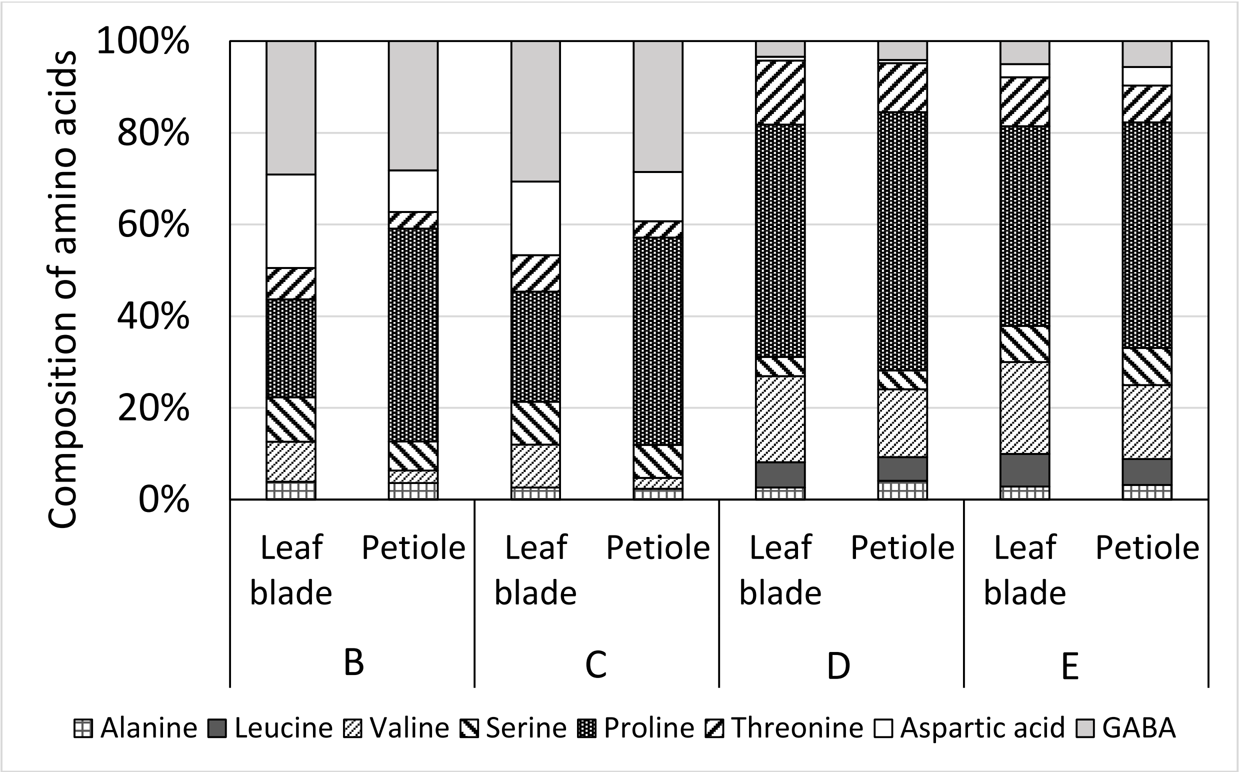

Figure S1. The composition of amino acids) in leaf blades and petioles of *G. biloba* treated with methyl jasmonate (JA-Me) on the adaxial and abaxial side of leaf. (**B**) control (lanolin),leaf samples collected on September 30; (**C**) JA-Me applied to the adaxial side, leaf samples collected on September 30; (**D**) JA-Me applied to the abaxial side, leaf samples collected on September 30; (**E**) naturally senesced leaf, samples collected on October 20.

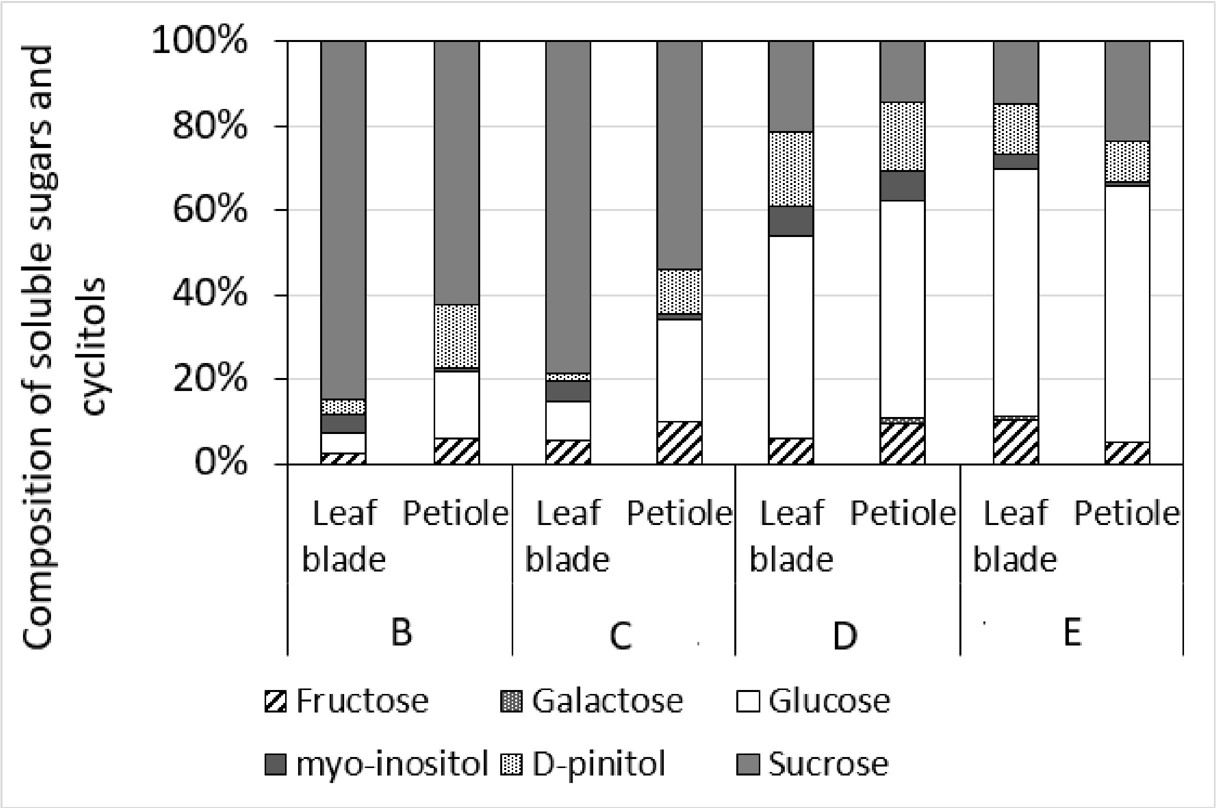

Figure S2. The composition of soluble sugars and cyclitols in leaf blades and petioles of *G. biloba* treated with methyl jasmonate (JA-Me) on the adaxial and abaxial side of leaf. (**B**) control (lanolin), leaf samples collected on September 30; (**C**) JA-Me applied to the adaxial side, leaf samples collected on September 30; (**D**) JA-Me applied to the abaxial side, leaf samples collected on September 30; (**B**) naturally senesced leaf, samples collected on October 20.
